# Supplementary material for: A Brief Executive Language Screen for Frontal Aphasia
Source: Brain Sci. 2021 Mar 10;11(3):353. doi: 10.3390/brainsci11030353 (PMC7998395; doi:10.3390/brainsci11030353)

Brief Executive Language Screen BELS

Materials: Stop watch. Mini-voice recorder (record all in 1 file)

Patient ID:	Lesion:	***Healthy Control***:
Date:	Pathology Type/Location:	
Age/DOB:	Handed: R/L	
Education Yrs:	Occupation:	
Learning Disability: Y/N	First Language:	

----

1. ***Spontaneous speech***: Description of Complex Scene (OZ Beach Scene – Page 7).

"*Describe everything you see going on in this scene*" Record full response. Note time. (Max=1min). DO NOT PROMPT SPECIFICS – OK to ask 'Anything else' once.

2. ***Rate Clinical characteristics of speech***: (tick the quality that best fits or if error type)

fluency: adequate__ reduced (<50 words/minute)__

prosody: normal__ abnormal__

errors: None__ semantic__ phonological__ syntax__

3. ***Spontaneous Speech - GOAL Version: Cookie Theft***

"*Talk continuously for 1 minute about what you see going on in this picture*" Record full response. Note time. (Max=1min). DO NOT PROMPT SPECIFICS – OK to ask 'Anything else' once.

4. ***Oral Apraxia:*** *"Ask the person to do the following:"*

Score 2 = Correct; 1 = Effortful or partial execution; 0 = unable to do

Cough
Blow out a match
Make a clucking sound
Whistle
Puff up cheeks

5. ***Sentence Repetition*** _____/5 Correct (Y/N) Errors

"Repeat this sentence"

	Correct (Y/N)	Errors
We enjoyed the movie		
It was very cold		
The shop was crowded		
It was a moonlit evening		
I like bananas		

6. ***Oral Naming*** __________/10 ***Repetition*** ______/10 ***Comprehension*** ______/10

Point to each picture (Page 8) and ask 'What is this?'

Say aloud each name and ask the person to: i. Repeat it (Repetition) and ii. point to it (Comprehension).

Mark correct for each or Note errors.

	Oral Naming	Repetition	Comprehension
Tuning fork	________________	________________	________________
Dolphin	________________	________________	________________
Harp	________________	________________	________________
Sunflower	________________	________________	________________
Saxophone	________________	________________	________________
Tiara	________________	________________	________________
Koala	________________	________________	________________
Wrench	________________	________________	________________
Caterpillar	________________	________________	________________
Celery	________________	________________	________________

7. ***Action Naming***__________/5 ***Past Tense Naming*** __________/5

"What is the action you see?" (Page 6) "Today I...; Yesterday I...?

	Action Naming	Past Tense Naming
shoot	________________	________________
bite	________________	________________
dig	________________	________________
drink	________________	________________
swim	________________	________________

8. *Word Fluency* (1 min): "Tell me as many words as you can that:

a. Begin with the letter 'S'; but no numbers, proper Nouns (e.g., seven or Susan) or using the same word but changing the endings. Try not to repeat words.
b. 'Animals' starting with any letter. Try not to repeat any items.
c/d. GOAL version: Give target number of items (20% higher than for standard): B; Fruit or vegetable

**a. S**	**b. Animals**	**c. GOAL = B**	**d. GOAL = Fruit or Vegetables**	
				15"
				30"
				45"
				60"
Total Number correct =				
Perseverations =				
Errors =				

9. ***Sentence Completion INITIATION Section***: HC = /5 LC = /5

"I am going to tell you a sentence that has the last word omitted. I would like you to say 1 word that completes the sentence meaningfully" Record response and note time from when you finish presenting the sentence until they start to respond (in seconds). If no response at 20 sec mark as error.

**HC**	The lecture should last about one...
**LC**	The Smiths had never visited that...
**LC**	The kind old man asked us to...
**HC**	I could not remember his...
**HC**	The paint turned out to be the wrong...
**LC**	The sun went down before we could...
**HC**	She went to the salon to colour her...
**LC**	Sometimes success is simply a matter of ...
**HC**	They sat together without speaking a single...
**LC**	He wondered if the storm would be ...

10. ***Sentence Completion INHIBITION Section***: HC = /5 LC = /5

"I am going to tell you a sentence that has the last word omitted. This time finish my sentence with **1 word completely unconnected to the sentence - that is a nonsense word.** Record response and note time from when you finish presenting the sentence until they start to respond (in seconds). If no response at 20 sec mark as error. Repeat instruction on 1st 2 items if they complete it meaningfully.

**HC**	The lecture should last about one...
**LC**	The Smiths had never visited that...
**LC**	The kind old man asked us to...
**HC**	I could not remember his...
**HC**	The paint turned out to be the wrong...
**LC**	The sun went down before we could...
**HC**	She went to the salon to colour her...
**LC**	Sometimes success is simply a matter of ...
**HC**	They sat together without speaking a single...
**LC**	He wondered if the storm would be ...

11. ***Luria Rhythm Tapping Task***

"*When I tap once, you tap once (Do this 2 times). When I tap twice, you tap twice (Do this 2 times). Now let's do this a few times*."

1-1-2-2-1-2-2

Score 1 = if could execute the run; 0 = unable to execute the run

"*Now we will do the opposite. When I tap once, you tap twice (do this 2 times). When I tap twice, you tap once (do this 2 times). Now we will do this a few times.*"

1-1-2-2-1-2-2

Do not proceed if they are unable to do the practice taps. Score 2 = able to execute the whole run; 1 = able to execute part of the run; 0 = unable to execute any of the run.

12. ***Memory:*** _______***/10***

"Can you tell me any of the 10 items that were on the card that you pointed to?"

Tuning fork _______________

Dolphin _______________

Harp _______________

Sunflower _______________

Saxophone _______________

Tiara _______________

Koala _______________

Wrench _______________

Caterpillar _______________

Celery _______________

13. ***Reading***:

NART ___ Number of Errors = ________ FSIQ equivalent________

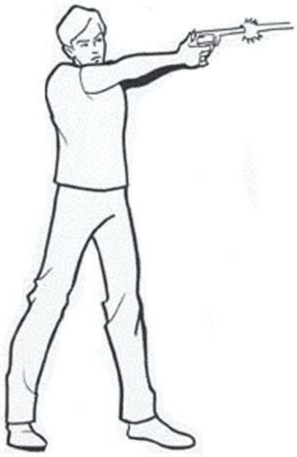

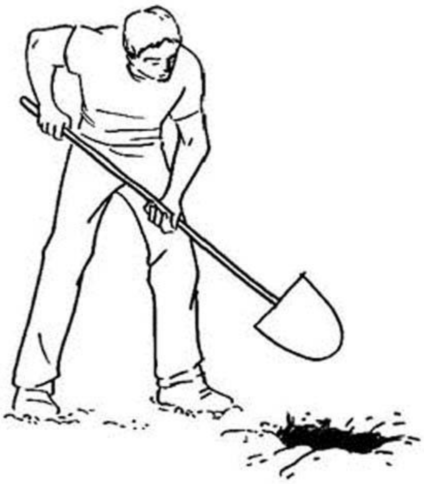

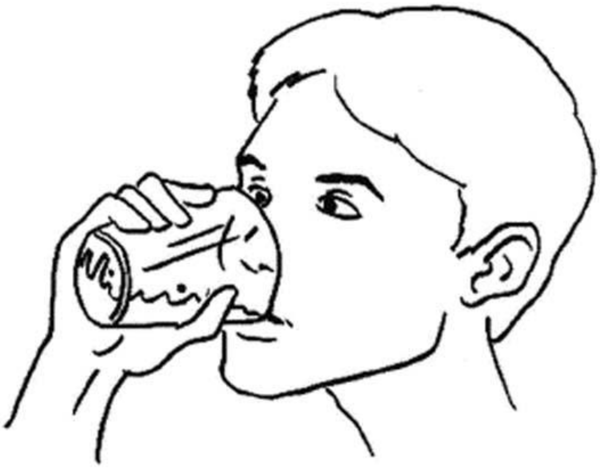

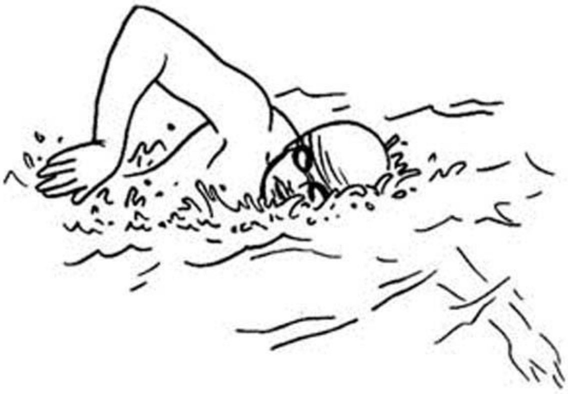

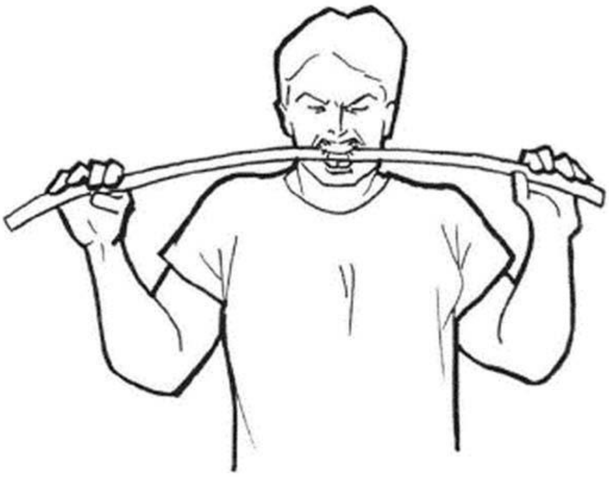

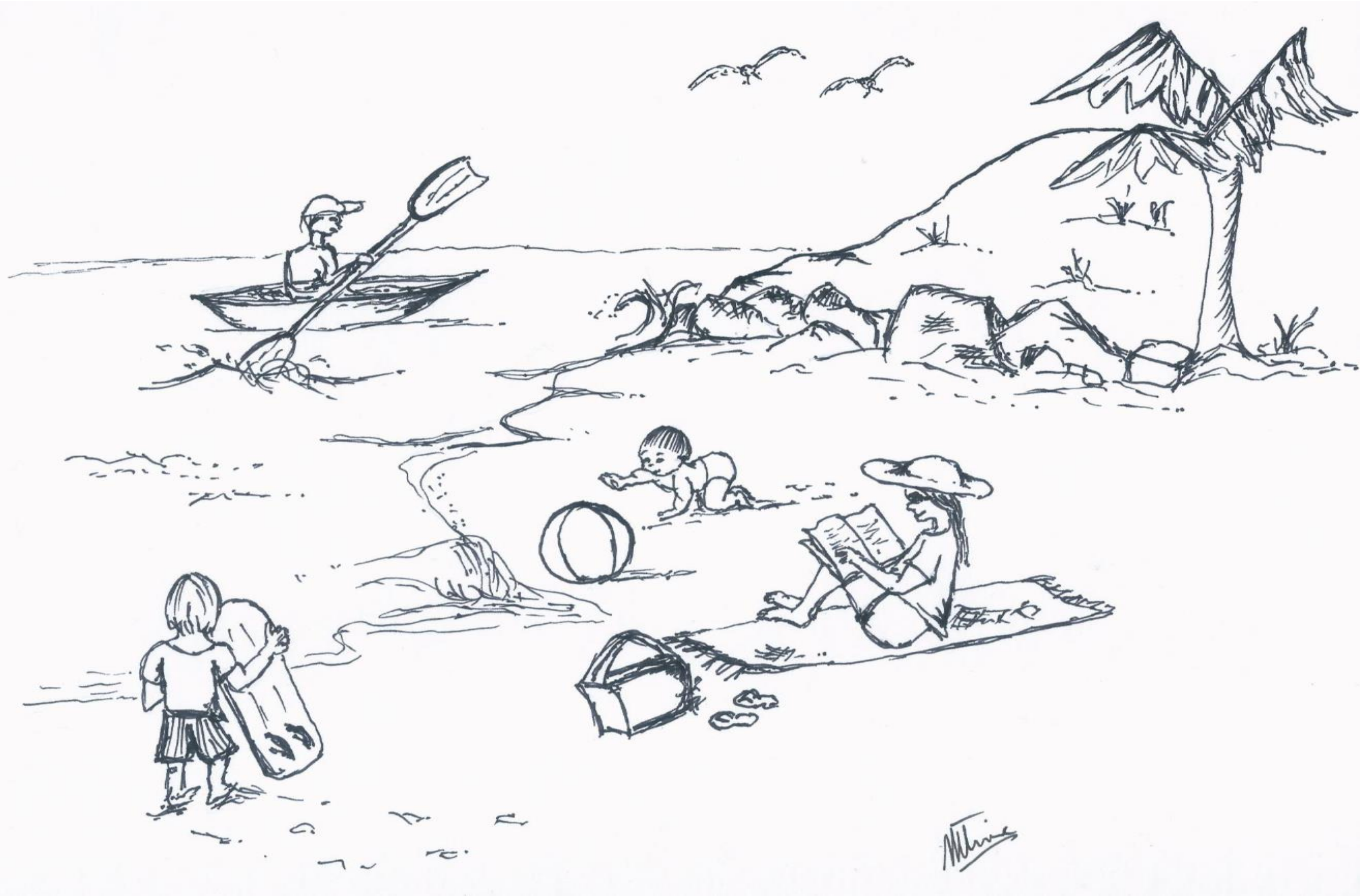

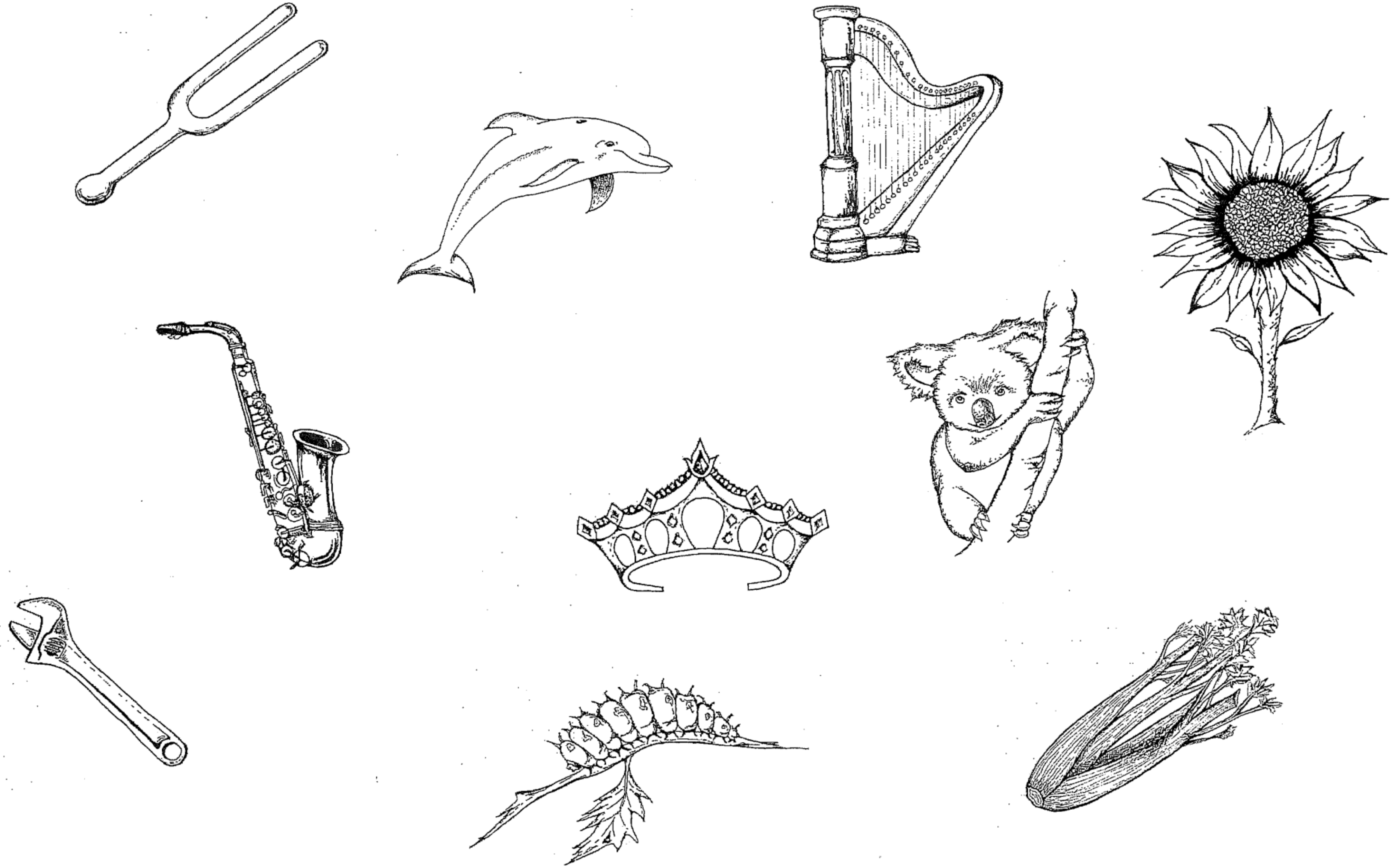

Supplement: Supplementary file 1 [file brainsci-11-00353-s001.pdf]
